# Pharmacy Practice and Education in Romania

**DOI:** 10.3390/pharmacy6010005

**Published:** 2018-01-08

**Authors:** Roxana Sandulovici, Constantin Mircioiu, Cristina Rais, Jeffrey Atkinson

**Affiliations:** 1 Faculty of Pharmacy, Titu Maiorescu University, 22, Dâmbovnicului, Sector 4, 040441 Bucharest, Romania; roxana.sandulovici@yahoo.com or farmacie@univ.utm.ro; 2 Faculty of Pharmacy, University of Medicine and Pharmacy “Carol Davila”, 6th Traian Vuia, Sector 2, 020956 Bucharest, Romania; constantin.mircioiu@yahoo.com (C.M.); cristina_rais@yahoo.com (C.R.); 3 Pharmacolor Consultants Nancy, 12 rue de Versigny, 54600 Villers, France

**Keywords:** pharmacy, education, practice, Romania, European Union

## Abstract

The PHARMINE (“*Pharmacy Education in Europe*”) project examined the organisation of pharmacy practice and education in the European Union (EU). An electronic survey was sent out to representatives of different sectors (community, hospital, industrial pharmacists, university staff, and students) in each individual EU member state. This paper presents the results of the PHARMINE survey on pharmacy practice and education in Romania. In the light of this data we examine to what extent harmonisation of practice and education with EU norms has occurred, whether this has promoted mobility of pharmacy professionals, academics and students, and what impact it has had on healthcare in Romania. The survey reveals the substantial changes in Romanian pharmacy practice and education since the 1989 change in government and Romania joining the EU in 2007. Romania remains, however, a poor country with expenditure on healthcare less than one-third of the EU average. This factor also impacts pharmacy practice. Although practice seems aligned with EU norms, this masks the substantial imbalance between the situation in the richer capital, Bucharest, and that of the poorer countryside. Harmonisation to EU norms in pharmacy education has not promoted student exchange and mobility but, rather, a brain drain in pharmaceutical graduates to other EU countries. Specialisation in industrial practice has been lost since 1989 with pharmacists being replaced by chemists. In hospitals the hospital pharmacist is being replaced by the clinical pharmacist.

## 1. Introduction

The PHARMINE (“*Pharmacy Education in Europe*”) consortium surveyed the state of pharmacy practice and education in the member states of the EU, including Romania, between 2008 and 2011, with an update in 2017. The methodology used and the principal results obtained have already been published [1]. 

PHARMINE gathered information on general (community) practice and specialisation in hospital and industrial practice. PHARMINE also dealt with assistant pharmacists—their education, training, and responsibilities were surveyed.

PHARMINE studied the administrative context of practice and education in the EU. In the EU pharmacy practice and education fall under two jurisdictions: national and European. The latter involves a confederal approach to decision-making. Freedom of movement, of residence, and of the right to work anywhere in the EU, is the cornerstone of the EU. In the case of pharmacy and other sectoral professions (nurses, midwives, doctors, dentists, architects, and veterinary surgeons) this is regulated by a system of automatic recognition of professional qualifications. To work in another EU member state, pharmacists apply to the relevant authority, providing proof of their qualifications. For sectoral professions, the EU issues directives which are ordinances laying down the broad imperatives [2]. An EU directive requires member states to achieve a particular result (in this case harmonisation of practice and education) without dictating the exact means of achieving that result. Directives leave the different member states with leeway to organise systems that are more or less harmonised with the EU paradigm. Thus member states may introduce national legislation relating to specialisation in practice and education, to ownership and management of pharmacies, etc.

In parallel to the EU directive, pharmacy education and training in Europe is also impacted by the Bologna agreement on the harmonization of degree courses and student and staff exchange [3]. The Bologna agreement, signed by the education ministers of the governments of the European Higher Education Area (48 members including the 28 EU member states), proposed several recommendations. In contrast to the EU directive Bologna recommendations are not legally binding. They include a degree structure for all university degrees with a bachelor (three years) followed by a master (two years). As with the EU directive, the idea behind the Bologna recommendations is to improve student mobility. However, the Bologna agreement and the EU directive do differ. The latter requires a five-year, “tunnel” degree structure for pharmacy, i.e., a degree course that offers no possibility for intermediate mobility after accomplishment of a three-year bachelor period. 

Mobility is also behind other aspects of the Bologna process: firstly, the development of tools to promote student exchange programmes, such as the European Credit Transfer and Accumulation System (ECTS). This provides credits to students for defined learning outcomes and their associated workload. Secondly, ECTS are coupled to a Diploma Supplement that describes the nature, level, context, content, and status of the studies that were successfully completed by the student at a given university. This system allows students to validate studies carried out at the host university by their home university. 

This paper looks at how the Bologna process has developed in Romanian universities. It is particularly interesting to examine how this affects pharmacy practice and education in a country that recently joined the EU in its fifth enlargement in 2007, following the 1989 revolution and the introduction of a multi-party, democratic government and free market measures. 

In order to place pharmacy within the general health context in Romania compared to the EU, it can be noted that in Romania life expectancy at birth (Table 1) is lower than (males) or equal to (females) the EU average of 79 years. Healthy life expectancy (EU average 70 years) is much lower. Furthermore, expenditure on health is less than one-third of the EU average ($3611 per capita). 

## 2. Design

Information was obtained from Romanian experts who replied to a questionnaire on:

- Pharmacy practice
  ⚬ Community, hospital, and industry
  ⚬ Organisation
  ⚬ Legislation
  ⚬ Education and training
- The adoption of the EU sectoral directive 2013/55/EU [2] in pharmacy practice and education
- The impact of the Bologna declaration [3]:
  ⚬ Organisation of the degree course with the existence or not of a bachelor/master structure,
  ⚬ Implementation of ECTS and the Erasmus programme on student and staff exchange [6].

The information is presented in the form of tables in order to facilitate legibility. This form of presentation was developed in association with this journal’s editorial board of directors and has been described in detail in a previous publication [7]. This presentation is aimed at easing the comparison of different EU countries as all country profiles are presented in the same tabular form.

## 3. Evaluation and Assessment

### 3.1. Organisation of the Activities of Pharmacists, Professional Bodies

Table 2 provides details of the numbers and activities of community pharmacists and pharmacies in Romania. Items are expounded in the “comments” column also include opinions and trends for the future.

Compared to the EU linear regression estimation (for definition and calculation see [1]), the ratio of the actual number of community pharmacists in Romania (per population) = 0.90. Thus, the number of pharmacists per population is slightly lower than the EU norm. The same comparison for community pharmacies produces a ratio of 0.90, again slightly below the EU norm. The activities and occupations of pharmacists in Romania are similar to those of community pharmacists in other EU member states. 

Globally, as far as pharmacy practice is concerned, Romania is close to EU norms and in line with the EU directive 2013/55/EU, thus, pharmacy practice is harmonised with that in other member states. However, behind the mean data there are highly non-homogeneous distributions. Thus, in Bucharest there are too many pharmacists and (almost) all small localities no longer have a pharmacy. There are various reasons behind this situation, but it is primarily connected to the low quality—especially economic—of life in villages. Furthermore, following the widespread emigration of young people to urban areas, only small numbers of old, poor people remain in the countryside so, economically, a pharmacy is no longer profitable.

Table 3 provides details of the numbers and activities of persons other than pharmacists working in pharmacies in Romania. 

The legal opportunity for pharmacy assistants to complete their education in order to become medical assistants for pharmacy is to be examined starting in 2017. Legislation in Romania tried to ensure compatibility of medical assistants for pharmacy with their European counterparts, but the practical consequences concerning the possibility of Romanian medical assistants for pharmacy to work elsewhere in the EU are unclear. As seen below, one aspect of harmonisation with the EU that is somewhat specific to Romania is the possibility that such harmonisation allows for Romanian pharmacy professionals to work elsewhere in the EU.

Table 4 provides details of the numbers and activities of hospital pharmacists in Romania.

In previous generations hospital pharmacy was connected to industrial pharmacy with the preparation, for example, of perfusions for hospital wards and many semisolid formulations. This type of hospital pharmacist is changing due to the low salary incentives in the national health service and the development of “clinical pharmacists”. These undergo specialisation in a 3-year internship “*rezidentiate*”. They are able to participate with the clinical team in the individualization of treatment of patients in a hospital. They act as clinical pharmacologists”. Thus in the future hospital pharmacists will become more clinical pharmacists.

Table 5 provides details of the numbers and activities of industrial pharmacists and pharmacists in other sectors in Romania.

Industrial pharmacists in Romania have similar practices and duties to those in other EU countries [1]. As accurate numbers of industrial pharmacists were not available for most European countries, a comparison with the EU average is not possible. 

Pre-1989, specialists in industry were pharmacists. The same was true in clinical laboratories in hospitals (“biochemistry laboratories”). After the liberalisation of community pharmacy practice, pharmacists migrated toward community pharmacies, where it was possible to obtain higher salaries. They were replaced by chemists in industry and by medical doctors in hospitals.

Pharmacists from the public laboratories for drug control (ICSMCF—Institute for State Control of Drugs and Pharmaceutical Research) disappeared following the disappearance of industrial and hospital laboratories in a purported harmonization with EU models. This had a negative impact on public control of the quality of drugs.

Table 6 provides information on professional associations for pharmacists in Romania.

### 3.2. Pharmacy Faculties, Students, and Courses

Table 7 provides details of pharmacy higher education institutions (HEIs), staff and students in Romania.

Compared to the EU average (for definition and calculation see [1]); the ratio for staff is 1.8 and, for students, 2.0. The student/staff ratio is around 2. It should be noted that a university post is not economically attractive compared to one in a community pharmacy. Furthermore, the number of students is decreasing and academic staff risk becoming jobless in the future. Tentative increases in student numbers include the French degree section at Cluj.

In Table 8 below are given the electives (courses open to choice) in the Romanian course.

Table 9 provides details of past and present changes in pharmacy education and training in Romania.

### 3.3. Teaching and Learning Methods

Table 10 provides details of hours by learning method (for further details on the definitions of the different methods see Reference [1]).

The degree is characterised by the substantial amount of hands-on training (65%).

### 3.4. Subject Areas

Table 11 provides details of student hours by subject area (for further details on the definitions of the subject areas see [1]). Student hours are presence hours, not student workload hours. 

Taking the MEDISCI/CHEMSCI ratio, medicinal sciences are still less than chemical sciences but, in the last 20 years, the proportion of medicinal sciences has increased continuously. It should also be noted that basic subjects (CHEMSCI, PHYSMATH, BIOLSCI) are concentrated in the early years, whereas more applied subjects (MEDISIC, PHARMTECH) in the later years. Traineeship is in the 5th year. Such chronological harmonisation is similar to that observed in other EU member states and should facilitate student exchange programmes.

### 3.5. Impact of the Bologna Recommendations [3]

Table 12 provides details the various ways in which the Bologna Declaration impacts on the pharmacy HEIs of Romania.

The application of the Bologna recommendations was subject to a long debate by the Council of Deans of the Faculties of Pharmacy of Romania in the 2004–2008 period. One argument was that young people have to enter earlier in their professional activity. A second argument was that in community pharmacy practice there is no longer a need for such detailed studies in chemistry, physics, and other fundamental sciences. This was evident in the PHAR-QA results [14]. The opinion of EU community pharmacists was that, for example, physics and analytical chemistry are no more than “quite important”. Furthermore, in the Romanian countryside, many pharmacies have only assistants with pharmacists dropping in from time to time. On this basis it could be argued that a three-year degree may be sufficient. The main argument against this is the specific nature of drug dispensation and the primordial element of patient safety.

Romania is in a somewhat unique situation relating to the harmonisation of pharmacy education and practice with other European countries in that harmonisation has led to emigration rather than exchange. In the last 10 years many young pharmacists from Romania left to work as pharmacists in several EU countries. Their numbers are increasing and approximately 3–4% of graduates currently leave Romania to work abroad. The phenomenon is still under control. This is different from the case of medical doctors who emigrated in large numbers such that Romania has, at the moment, an acute lack of medical personal. 

### 3.6. Impact of EU Directive 2013/55/EC [2]

Table 13 provides details the various ways in which the EC directive impacts on pharmacy education and training in Romania.

Romania mainly conforms to the different aspects of the EU directive with, notably, a tunnel degree. It should be noted that the directive clearly orients pharmacists toward community pharmacy practice reducing the possibilities for the development of pharmacy practice in clinical trials, industry, regulatory affairs, hospital pharmacy, etc.

## 4. Discussion and Conclusions

In essence, the survey reveals substantial changes in Romanian pharmacy practice and education since the 1989 change in government and Romania joining the EU in 2007. Some elements of the previous regime remain, such as the education of pharmacy assistants. Progress has been made towards harmonisation with the EU. Romania remains, however, a poor country with expenditure on healthcare at less than one-third of the EU average. This factor thwarts the impact of harmonisation on pharmacy practice. Thus, although practice seems aligned with EU norms, this masks substantial imbalance between the situation in the richer capital, Bucharest, and that of the poorer countryside. Harmonisation to EU norms in pharmacy education has not promoted student exchange and mobility but, rather, a brain drain in pharmaceutical graduates to other EU countries. Although this has not yet had the very serious consequences of emigration of medical general practitioners, the situation calls for remediate measures. Specialisation in industrial practice has been lost since 1989 with pharmacists being replaced by chemists. In hospitals the hospital pharmacist has been replaced by the clinical pharmacist. 

The question arises as to whether harmonization could lead to an improvement in the Romanian health system and, more generally, what can do pharmacists in this respect? As seen above, harmonisation with EU norms is not always positive in that this can lead to pharmacy professionals emigrating to other countries. Another aspect of this concerns the prescription of generic drugs. This accounts for 88% of all prescriptions in the USA and approximately 20% in Romania [15]. Several causes are involved here including not only government economic policy but also the diminution in the competences of industrial pharmacists in areas such as bio-pharmacy and pharmacokinetics, good manufacturing practice, good clinical practice, and quality assurance systems, etc.—essential information for a clear understanding that bioequivalence implies therapeutic equivalence.

Finally the general context is that Romania is a poor country, with worse health conditions in comparison with other European countries. This situation is generated by many causes but one of these is clearly connected with education in the medical and pharmaceutical domain. Given this situation harmonization with EU norms was considered as a way for improving the actual situation. Harmonization started with the EU directives, first of all with curricula and, particularly, with the ratio between theoretical and practical education. The most important consequence of this legislative harmonization was the recognition of diplomas obtained from Romanian faculties of pharmacy and opportunity for pharmacists to work practically in all European countries. Unfortunately, this did not have the expected impact on student and staff exchange in other EU member states. Another negative effect was the alignment of EU directives with the most basic levels of knowledge and education. This promoted ignorance of new disciplines, such as information technology, bio-pharmacy, advanced pharmacokinetics, clinical pharmacy, etc., thus proving a bad model for Romania.

The final conclusion is that harmonization with EU norms is a long, ever-evolving process which is to be pursued in parallel with in-depth analysis, starting from local traditions and institutions, of all possible consequences. 

## Figures and Tables

**Table 1:** Health statistics for Romania [4,5].

Total Population	19,511,000
Life expectancy at birth m/f (years)	71/79
Healthy life expectancy at birth m/f (years)	59/59
Total expenditure on health per capita	$1074

**Table 2:** Numbers and activities of community pharmacists and pharmacies in Romania.

Item	Numbers	Comments
Pharmacists	*Circa* 13,600	3533 work in Bucharest and 10,067 elsewhere 1435 inhabitants/community pharmacist (Bucharest 525 inhabitants/community pharmacist) EU average: 2145 [1]
Pharmacies	5938	Inhabitants/pharmacy: 3286. EU average: 4407 [1] Pharmacists/pharmacy: 2.3. EU average: 2.1 [1]
Competences and roles of community pharmacists		The competencies of community pharmacists are: Supplying prescription medicines Managing medicines for some ailments Giving advice on medicines creening, diagnostic services
Is ownership of a community pharmacy limited to pharmacists?	No	The community pharmacies are private institutions. Their owners need not be pharmacists as long as they hire a pharmacist as the manager.
Rules on geographical distribution of pharmacies?	Yes	Demographic criteria only [8,9]. Bucharest: one pharmacy per 3000 inhabitants cities that are capital of their respective district: one pharmacy per 3500 inhabitants other cities: one pharmacy per 4000 inhabitants Exceptions from these provisions are the community pharmacies found in railway stations, airports, and in large surface commercial centres.
Are drugs and health care products available to the general public by channels other than pharmacies?	Yes	Through stores that sell plants or medicines from plants and OTC (*Plafar*) only. Recently e-pharmacies have appeared, but their legal status is not yet clearly established. Despite the fact that present legislation imposes a series of conditions for the way pharmacies work and how they sell drugs, these do not directly restrict marketing them online. According to a draft normative act, online marketing for OTC drugs will be allowed for authorised pharmacies only, with the goal to reduce the risks to which the online buyers are exposed at present.

**Table 3:** Numbers and activities of other personnel working in pharmacies in Romania.

Item	Numbers	Comments
Are persons other than pharmacists involved in community practice?	Yes	There are two types of assistants working in pharmacies: - pharmacy assistants have three years’ education in a postsecondary school (technical college). This represents the pre-1989 education system survived and is still predominant. - medical assistants for pharmacy follow a three-year course in a university faculty of pharmacy [10]. This second category was introduced in 2004 in order to assure recognition in Europe. In Romanian pharmacy practice no difference is made between the two categories and both are also called “pharmacy assistants”. They perform dispensing and counselling of drug products, under the guidance of a pharmacist.
Their numbers and status	>120,000	
Organisations providing and validating education and training of the 3-year courses		The curricula of postsecondary schools was up-graded and extended by university pharmacy faculties for the education of medical assistants for pharmacy starting from 2004 onwards, and validated by Romanian Agency for Quality of University Education (ARACIS) [11]. In a second phase the Ministry of Education and the Ministry of Health imposed the same curricula on post-secondary schools. Thus practically the structure is essentially similar with some quantitative differences. Entrance exams and final evaluations are similar, but they receive a different diploma [12].
Subject areas		Fundamental disciplines: human anatomy, physiology, physiopathology, microbiology, clinical pathology, cellular and molecular biology, general and inorganic chemistry, organic chemistry, mathematics, computer sciences, Applied disciplines: chemical bases of the drug (inorganic and organic chemistry), physical-chemical bases of drug formulation, medicinal plants, descriptive and metabolic biochemistry, instrumental analysis techniques, medical semiology/clinical pathology, first-aid, pharmaceutical and medical terminology, elements in nutrition, bioethics and ethics, Specialty disciplines: drug analysis, the bases of pharmaceutical techniques, therapeutic chemistry, communication in pharmacy, elements of industrial pharmaceutical technology, elements of toxicology, pharmacology, phyto-therapy, notions of pharmacovigilance, cosmetic products, para-pharmaceutical and medical products, vegetal products, dietary supplements), Complementary disciplines: medical information technology, modern languages, scientific research methodology, elements of pharmaceutical management and marketing.
Competences and roles		Dispensing and counselling of: Medical assistants: OTC medicines and plant products Pharmacy assistants: drug products

**Table 4:** Numbers and activities of hospital pharmacists in Romania.

Item	Numbers	Comments
Does such a function exist?	Yes	
Number of hospital pharmacists	692	One hundred and twenty in Bucharest (17%) and 572 in the rest of the country. Hospital pharmacies are small with 1–2 pharmacists and 2–3 medical assistants. The number of specialists is insufficient to cope with the demands of the numerous patients hospitalized. Therefore it is necessary to increase the numbers of staff for the development of patient care. The lack of specialist staff is also due to the low salaries in hospitals.
Number of hospital pharmacies	564	One hundred and twenty in Bucharest (21%) and 444 in the rest of the country.
Competences and roles of hospital pharmacists		These are similar to those in other EU member states [1].

**Table 5:** Numbers and activities of industrial pharmacists and pharmacists in other sectors in Romania.

Item	Numbers	Comments
**Industrial Pharmacy and Pharmacists**		
Number of pharmacists working in industry	Around 120	
Competences and roles		Synthesis and production of chemical entities and drugs Research and development, including formulation and control of drug systems, evaluation of bioavailability of active substances Cooperation in preclinical drug evaluation (safety and efficacy) Cooperation in clinical trials (safety and efficacy) Quality assurance of production Registration of drugs Marketing Distribution
**Pharmacists working in other sectors**		
	100–200	
Sectors in which pharmacists are employed		Clinical Trials, Armed Forces, National Medicinal Agency

**Table 6:** Professional associations for pharmacists in Romania.

Item		Comments
Registration of pharmacists	Yes	The National College of Pharmacists has colleges in each district, including Bucharest [13]. All the branches are active in registration of pharmacists. After graduation, in order to work as a pharmacist, the candidate must obtain the Pharmacists’ Membership Certificate from the National Pharmacy College. Further continuous educational training is required—pharmacists have to accumulate 40 continuous education credit points/year in order to carry on with their pharmacy practice.
Creation of pharmacies and control of territorial distribution	Yes	A dossier has to be presented in order to open a new community pharmacy. This contains information on: The registration number from the Trade Register of the new commercial society created The personal employed (professional qualification, number) The work programme The proof of ownership The proof of the demographic criteria. This must be sent to the Ministry of Health, Department of Strategies and Medicine Politics. After verification of legal criteria, inspections are performed, by the Ministry of Health and the College of Pharmacists, in order to release the authorisation to function.
Ethical and other aspects of professional conduct	Yes	Romania has a Code of Ethics for pharmacists approved by the General Assembly of Pharmacists in 2009 [13].
Quality assurance and validation of university courses	Yes	

**Table 7:** Pharmacy higher education institutions (HEIs), staff, and students in Romania.

Item	Number	Comments
Number of pharmacy HEIs in Romania	11	West University *Vasile Goldis* Arad, http://www.uvvg.ro/site/; http://www.4icu.org/reviews/12323.htm University of Medicine and Pharmacy *Carol Davila*, Bucharest, http://www.ceebd.co.uk/ceeed/un/rom/ro003.htm University of Medicine and Pharmacy Cluj Napoca, http://www.ceebd.co.uk/ceeed/un/rom/ro020.htm; http://www.umfcluj.ro/ Ovidius University, Constanta, http://www.euroeducation.net/euro/ro025.htm; http://www.univ-ovidius.ro/ University of Medicine and Pharmacy Craiova, http://www.umfcv.ro/en/index.html University of Medicine and Pharmacy Iassy, https://www.medicaldoctor-studies.com/study-medicine-abroad/study-medicine-in-romania/university-of-medicine-pharmacy-in-iasi/ Oradea University, http://www.uoradea.ro/ University of Medicine and Pharmacy Targu Mures, http://www.euroeducation.net/euro/ro048.htm; http://www.umftgm.ro/ University of Medicine and Pharmacy Timisoara, http://www.umft.ro/ University Lower Danube, Faculty of Medicine and Pharmacy, Galati, http://www.ugal.ro/ Titu Maiorescu University, Faculty of Pharmacy, Bucharest, http://www.utm.ro/en/
Public pharmacy HEIs	2	West University *Vasile Goldis* Arad Titu Maiorescu University, Faculty of Pharmacy, Bucharest
Independent faculty	Part of “Medicine & Pharmacy” HEIs	University of Medicine and Pharmacy *Carol Davila*, Bucharest University of Medicine and Pharmacy Cluj Napoca University Ovidius, Constanta University of Medicine and Pharmacy Craiova University of Medicine and Pharmacy Iassy University Oradea, University of Medicine and Pharmacy Targu Mures University of Medicine and Pharmacy Timisoara
Faculty attachment	Attached to a medical faculty	Faculty of Medicine, Dentistry and Pharmacy, at the “University Vasile Goldis” Arad Faculty of Medicine and Pharmacy, Galati Faculty of Pharmacy, Titu Maiorescu University, Bucharest
Do HEIs offer B and M degrees?	No	All pharmacy schools have a five-year integrated system. There is no split between the first years 1–3 and years 4–5, and there is no “diploma” that gives the right to work after completing the first three years.
**Teaching staff**		
Staff	Around 100 at each HEI = *circa* 1000 in all	There is no national database of teaching staff in pharmacy.
Professionals from outside the HEIs, involved in E&T	Around 3% of staff	The professionals from outside the HEI’s involved in education and training are community pharmacists in charge of the traineeship period, researchers from hospitals or research units.
**Students**		
Number of places on entry following secondary school	Around 50–250 per HEI	Around 50–200 students for smaller faculties, and 250 for Bucharest and Cluj, together with 50–100 places for training in English or French languages.
Number of applicants for each entry place	100–400 per HEI	Around two applicants per place, but the number of candidates diminishes in time following massive emigration of the young population to other countries.
International students (EU)	2%	Students from Greece, Bulgaria.
International students (non EU)	20%	Students from: Albania, Jordan, Iraq, Iran, Israel, Lebanon, Macedonia, Moldavia, Mongolia, Morocco, Nigeria, Palestine, Syria, Tunisia.
Specific pharmacy-related entrance examination.	Yes	Botany or anatomy and organic chemistry—subjects with potential application in pharmacy.
Other form of entry requirement at a national level	Yes	Graduates who already have a degree from other faculties (medicine, chemistry, biology) and want to obtain a pharmacy degree can start on advanced entry, 2nd or the 3rd year based on ECTS.
Graduates that become registered pharmacists.		Difficult to establish given drop-out rate.
**Advanced entry**		
Entrance after a first bachelor year.	no	
**Fees per year**		
For home and EU students		9000 RON (*circa* €2000)
For non-EU students		€6000

**Table 8:** Specialisation electives in pharmacy HEIs in Romania.

Item		Comments
Do HEIs Provide Specialised Courses?	Yes	In order to work in industry or a hospital as an executive it is sufficient to be a graduate of the integrated five-year programme. One can become a “Qualified Person” after two years’ activity in certified (Good Manufacturing Practice, Good Clinical Practice, Good Laboratory Practice) industrial units in the field of qualitative analysis of the medicines, quality control of active substances, or any other tests required to check the quality of medicines. The certification is validated by the National Medicines Agency after evaluation of the activity of the candidate. It is not mandatory to be a pharmacist in order to become a Qualified Person. In order to obtain a leading position in a hospital pharmacy it is necessary to become a “specialist” following two years’ education in the framework of “*rezidentiate*” in a faculty of pharmacy and a final exam. Only a small number of hospitals have pharmacists specialized in clinical pharmacy. Most of them are “specialists” in community pharmacy.

**Table 9:** Past and present changes in education and training in Romania pharmacy HEIs.

Item	Comments
Have there been any major changes since 1990?	The change in the governmental regime in 1989 and the preparation for joining the EU in 2007, promoted harmonisation with EU pharmacy practice and education. Other forces were at work. Privatization of pharmacies induced a substantial increase in the number of pharmacies leading to an increase in the number of faculties of pharmacy (from four to 11) and ten times the number of students. Following a drastic reduction in the preparation of medicines in the pharmacy, the preponderance of chemical sciences disciplines has diminished in favour of medical disciplines in the pharmacy degree.
Are any major changes envisaged before 2019?	Two factors: (1) the decrease in the number of young people in Romania, and (2) the increase in emigration of pharmacists to other EU countries, impose an in-depth analysis of the aims and methods of pharmacy education and training, based on the competences required by the labour market and future practice.

**Table 10:** Student hours by learning method.

Method	Year 1	Year 2	Year 3	Year 4	Year 5	Total	%
Lecture	308	336	350	350	238	**1582**	32.4
Tutorial + Practical	434	462	490	434	266	**2086**	42.8
Project	0	0	0	0	60	**60**	1.2
Traineeship (community or hospital pharmacy)	60	60	60	120	780	**1140**	22.1
Electives + Optional	14	14	14	14	14	**70**	1.4
**Total**	816	872	914	918	1358	**4878**	100

**Table 11:** Student hours by subject area.

Subject Area	Year 1	Year 2	Year 3	Year 4	Year 5	Total	%
CHEMSCI	238	238	238	168	0	882	20.1
PHYSMATH	238	0	0	56	0	294	6.7
BIOLSCI	154	14	336	14	14	532	12.1
PHARMTECH	28	14	84	140	210	476	10.8
MEDISCI	0	182	98	392	168	840	19.1
LAWSOC	14	14	0	42	56	126	2.9
GENERIC + TRAINEESHIP	172	172	60	60	780	1244	28.3
Total	844	634	816	872	1228	4394	100

CHEMSOC: chemical sciences; PHYSMATH: physical and mathematical sciences; BIOLSCI: biological sciences; PHARMTECH: pharmaceutical technology; MEDISCI: medicinal sciences; LAWSOC: law and social sciences; GENERIC: generic competences.

**Table 12:** Ways in which the Bologna Declaration impacts on Romanian pharmacy HEIs.

Item	Comments
“Comparable degrees with diploma supplement”	The degree structure is comparable to that observed in other EU member states (see above). A diploma supplement is delivered according to European directives (it is both in Romanian and English).
“Two main cycles (B and M) with entry and exit at B level”	There is a five-year integrated course with no possibility of graduation after three years
“European Credit Transfer System (ECTS) system of credits with links to life-long learning (LLL)”	Theoretically, this system was accepted and formally adopted in 1998. Practically, it was applied step-by-step. The transfer of credits was accepted between faculties of pharmacy and later between faculties of pharmacy, medicine and chemistry, all inside Romania. The acceptance of credits from foreign universities is discussed case-by-case.
“Addressing obstacles to mobility”	Both language barriers and lack of financial support. Only incoming students receive language tuition.
European/international quality assurance of courses	Maybe in the near future. Pharmacy courses and traineeship are validated by the Ministry of Education and the Romanian Agency for Quality Assurance in Higher Education (ARACIS [11]).
European dimension	Our staff was involved in European Projects: Cooperation in Science and Technology (COST) Joint Research Center (JRC) Ispra PHAR-QA the follow-on from PHARMINE
ERASMUS staff exchange to Romania from elsewhere	Rare
ERASMUS staff exchange from Romania to other HEIs	Not frequently
ERASMUS student exchange to Romania from elsewhere	Less than 5 students/year
ERASMUS student exchange from Romania to other HEIS	Number of student months: 3–6 2 students in 2008, 3 students in 2009, all to Italy

**Table 13:** Ways in which the elements of the EC directive (left column) impact on Romanian pharmacy HEIs.

Item	Comments
“Evidence of formal qualifications as a pharmacist shall attest to training of at least five years’ duration, …”	This applies.
“…four years of full-time theoretical and practical training at a university or at a higher institute of a level recognised as equivalent, or under the supervision of a university.”	Yes, applied ad literam (4.5 years of full time theoretical and practical training and six months traineeship in a hospital or community pharmacy). Professors from the pharmaceutical technology department validate the traineeship through an oral/written examination in which the student must solve a problem in pharmaceutical technology (e.g., a pharmaceutical preparation). At the end of this period, the student must also present a notebook with his/her activity in the practice period and be able to answer questions regarding pharmaceutical practice.
“…six-month traineeship in a pharmacy which is open to the public or in a hospital, under the supervision of that hospital’s pharmaceutical department.”	Industrial traineeship is allowed in a community or hospital traineeship, but for only one of the six compulsory months
